# Bilateral changes in tendon structure of patients diagnosed with unilateral insertional or midportion achilles tendinopathy or patellar tendinopathy

**DOI:** 10.1007/s00167-019-05495-2

**Published:** 2019-04-01

**Authors:** Lucas Maciel Rabello, I. van den Akker-Scheek, Ireen F. Kuipers, R. L. Diercks, Michel S. Brink, J. Zwerver

**Affiliations:** 1 Department of Sports and Exercise Medicine, University of Groningen, University Medical Center Groningen, Hanzeplein 1, 9713 GZ Groningen, The Netherlands; 2 Department of Orthopedics, University of Groningen, University Medical Center Groningen, Hanzeplein 1, 9713 GZ Groningen, The Netherlands; 3 Center for Human Movement Sciences, University of Groningen, University Medical Center Groningen, Antonius Deusinglaan 1, 9713 AV Groningen, The Netherlands

**Keywords:** Tendinosis, Jumper’s knee, Imaging, Tendinitis, Enthesiopathy

## Abstract

**Purpose:**

Changes in tendon structure are commonly seen in patients with unilateral achilles (AT) or patellar (PT) tendinopathy but might also be present on the asymptomatic side, indicating a higher risk for developing symptoms. The aim of this study is to compare tendon structure of the symptomatic side with the asymptomatic side in AT and PT patients and control subjects.

**Methods:**

A total of 46 patients with unilateral AT (16 insertional and 30 midportion) and 38 with unilateral PT were included. For the control group, a total of 18 Achilles tendons and 25 patellar tendons were scanned. Tendon structure was assessed using ultrasound tissue characterisation (UTC), which quantifies tendon organisation dividing the structure into four different echo types (I–IV).

**Results:**

There were significant differences in echo types I, III, and IV between symptomatic and asymptomatic sides and controls. Additionally, there was a significant difference between the symptomatic and the asymptomatic side for all tendinopathy locations. In the insertional AT tendon portion, the symptomatic side showed a higher percentage of echo type III. For the midportion AT, the symptomatic side showed a lower percentage of echo type I and a higher percentage of echo types III and IV. For the patellar tendon, the symptomatic side showed a higher percentage of echo types III and IV. All differences were higher than the minimal detectable changes.

**Conclusion:**

Although patients have symptoms unilaterally, the tendon structures are compromised on both sides. These results stress the importance of monitoring both symptomatic and asymptomatic tendon structures and in addition highlight that the asymptomatic side should not be used as reference in clinical practice.

**Level of evidence:**

III.

## Introduction

Tendinopathy is a common injury that causes pain and functional limitations [19] and leads patients to seek medical attention. In the Dutch general practice population, the prevalence of Achilles and patellar tendinopathy is 2.35 and 1.60 per 1000 person-years, respectively. The diagnoses of AT and PT are based mainly on clinical examination [18, 25], although imaging might be used to exclude other musculoskeletal disorders or to help clinicians with their treatment decision [20].

It is well-known that imaging abnormalities are present in tendons diagnosed with tendinopathy [7, 37]. An increase in tendon cross-sectional area (CSA) and anteroposterior (AP) diameter and the presence of hypoechoic areas are some of the abnormalities seen during ultrasound (US) and/or magnetic resonance imaging (MRI) examination [7, 37]. However, these abnormalities can also be present on the asymptomatic side [7, 14, 16, 23]. These findings are mainly based on conventional US examination, which has the disadvantage of subjective interpretation of images (normal or abnormal tendon), with a limited objective quantification of the tendons’ dimensions [11]. Investigations of the changes in tendon structure on both the symptomatic and asymptomatic sides using quantitative imaging techniques such as ultrasound tissue characterisation (UTC) are almost completely lacking. The advantage of this tool, compared to conventional US, is that it captures a three-dimensional ultrasound image of the tendon and uses standardised parameters (transducer tilt angle, depth, and gain settings) [11]. UTC quantifies the consistency of intensity and distribution of gray levels of images of the tendon using a computer algorithm. Based on this, four echo types (I–IV) are created for every transverse image and expressed in percentages. Echo type I is the most stable echo pattern and echo type IV the least stable [33].

Investigating tendon structure abnormalities is important because these are considered a risk factor for developing symptoms [23]. Knowledge about both symptomatic and asymptomatic tendon structure is, therefore, relevant for monitoring changes due to adaptation to load during rehabilitation programs. To the best of our knowledge, only one study, by Docking et al., compared the structure of asymptomatic to symptomatic and control tendons using UTC [12]. Their previous study focused on the Achilles tendon and found that the structure of the symptomatic side was worse than the asymptomatic side [12]. However, when comparing symptomatic patients with controls they also observed that not only the structure on the symptomatic side was abnormal, but that the structure on the asymptomatic side was also affected. These findings do not support clinical practice, where both sides are usually examined and conclusions and management are often based on this comparison.

No previous study has investigated patients diagnosed with AT differentiating between insertional and midportion symptoms. The fact that the tendon insertion has a primary function of dissipating stress and shows a different tendon structure [4, 32, 40] stresses the importance of conducting separate analyses for patients diagnosed with insertional and midportion AT. Moreover, such changes in the patellar tendon have not been investigated. Additionally to the tendon investigated, no previous study has recruited a representative sample of tendinopathy patients seen by physicians in their clinical practice in a hospital setting. In addition, in daily practice clinicians often use the asymptomatic side as normal reference during the examination of patients with unilateral tendinopathy. It seems relevant to compare results to those of young healthy controls as a reference group, to draw definite conclusions about normal and abnormal tendon structure.

Thus, the aim of this study is to quantify and compare the tendon structure of the symptomatic side with the asymptomatic side in a large representative sample of patients diagnosed with insertional or midportion AT or PT using UTC. Secondary aim is to compare these results with those of young controls subjects without tendon complains.

## Materials and methods

Retrospective cross-sectional study with patients diagnosed with AT or PT. The protocol was approved by the medical ethical committee of the University Medical Center Groningen (M19.227797) and all patients signed an informed consent allowing access to their medical files.

### Subjects

Between 2015 and 2017 a total of 151 patients with Achilles (82 patients) or patellar (69 patients) tendon complaints visited the Tendinopathy Clinic at the University Sports Medicine Center, located at the University Medical Center Groningen (UMCG), the Netherlands. Patients were referred to the clinic by their general practitioner (GP) or by a medical specialist. Inclusion criteria were being diagnosed with unilateral insertional or midportion AT or PT and having symptoms longer than 3 months. Patients with bilateral symptoms and/or who had received injections in the tendon previously and/or had surgery in the lower limbs were excluded. All patient and injury information was collected using medical records.

To rule out the effect of age on tendon structure, young healthy subjects without any history of tendon complaints or diagnosis of tendinopathy in the lower limbs were recruited from the general population to serve as control group.

### Tendinopathy diagnosis

All patients underwent a clinical examination, which included an interview and physical examination, performed by a sports physician (JZ) specialised in tendon rehabilitation. In addition to it, patients completed the Dutch version of the Victorian Institute of Sport Assessment (VISA) questionnaire [30, 34, 38, 42]. This instrument includes questions about pain, function, and sport activity and is considered a valid and reliable index of the severity of Achilles or patellar tendinopathy [30, 38]. The majority of the patients were referred to the radiologist for an imaging examination (Radiographs, US and/or MRI) to exclude other pathologies and increase the likelihood of a diagnosis of AT or PT.

Insertional AT was defined as pain and symptoms at the insertion of the Achilles tendon to the calcaneus (up to 2 cm). Midportion AT was defined as pain and symptoms in the main body of the Achilles tendon, i.e., 2–6 cm proximal to the insertion. PT was defined as pain and symptoms at the insertion of the patellar tendon up to the inferior pole of the patella.

### UTC imaging examination

After diagnosis, patients underwent a UTC examination as a part of the usual care. Bilateral scans were performed by a single examiner with experience performing UTC scans (LMR). Images were acquired using a 7–10 MHz linear ultrasound transducer (SmartProbe 12L5-V, Terason 2000+; Teratech; Burlington, MD, USA) positioned in a tracking device (UTC Tracker, UTC Imaging, Stein, The Netherlands) that moved automatically along the tendon long axis over a distance of 12 cm recording regular images at intervals of 0.2 mm. Transducer tilt, angle, gain, focus and depth were standardised by the tracking device. Images from the sagittal, coronal and transverse planes were compiled to create a 3D view of the tendon. The UTC imaging tool can be considered a reliable tool to investigate the Achilles tendon (excellent [0.92–0.95] inter-observer reliability) [34] and patellar tendon structure (fair to good inter [0.46–0.83]- and intra-observer [0.61–0.88] reliability) [van Ark et al. personal communication].

A standardised protocol was used to perform the UTC scan of the Achilles and patellar tendons. The Achilles tendon was scanned with the patient placed prone on the examination table with feet hanging over the edge and with the ankle in maximal passive dorsiflexion [17]. The patellar tendon was scanned with the patient positioned supine on the examination table with the knee positioned in an angle of approximately 100° flexion. The tracking device was placed perpendicular to the long axis of the tendon (achilles or patellar), ensuring that the calcaneus or the inferior pole of the patella was observed on the imaging device. Further, this bony landmark served as reference point to analyze tendon structure. The Achilles and patellar tendons were scanned from distal to proximal and from proximal to distal, respectively. Coupling gel between transducer, standoff pad and skin was applied to ensure maximum contact. The recorded images were stored using UTC software (UTC v 1.05, UTC Imaging). The UTC algorithm quantified echo types across a rolling window of 17 continuous images.

Tendon structure was quantified from a region of interest (ROI) that was selected around the border of the tendon in the sagittal plane. For both Achilles and patellar tendons, ROIs were contoured at intervals of 5 mm. For patients with insertional Achilles tendinopathy, five ROIs were contoured from the proximal border of the calcaneus to 2 cm proximally. In patients diagnosed with midportion tendinopathy, nine ROIs were contoured from 2 cm above the calcaneous to 6 cm proximally. For those patients diagnosed with patellar tendinopathy five ROIs were contoured from the apex of the patella to 2 cm distally.

Using UTC, tendons were divided into four valid echo types: echo type I, intact and aligned tendon bundles; echo type II, less integer and waving tendon bundles; echo type III, mainly fibrillar tissue; echo type IV, a mainly amorphous matrix with loose fibrils, cells or fluid [33].

### Statistical analysis

Descriptive statistics with means and standard deviations (SD) were used. Non-parametric tests were used to compare the UTC results since the data were not normally distributed. The related-samples Wilcoxon signed rank test was used to compare the symptomatic side with the asymptomatic side for all echo types (I–IV). The comparison between symptomatic and asymptomatic sides with controls was performed using the independent samples Mann–Whitney *U* test. A Bonferroni correction was applied to adjust for multiple testing; the alpha level was set at 0.01.

To determine whether the found changes are larger than the minimal detectable changes (MDC), first the MDC values had to be determined for each echo type, as this was not done in earlier studies. Therefore, a test–retest reliability study was performed in a separate sample of 20 tendons (10 achilles [insertion and midportion] and 10 patellar) of patients with similar demographics as the study population. Tendon structure was investigated using the same methods previously described in this manuscript. A two-way mixed single-measures intra-class correlation coefficient was reckoned to calculate standard error of measurement [SEM = Standard deviation × √(1 − ICC)]. To calculate MDC the following formula was used: MDC = 1.96 × SEM × √2. All analyses were performed using IBM SPSS (version 22). All patients that visited the tendinopathy clinic from 2015 to 2017 and met the inclusion/exclusion criteria were included.

## Results

**Fig. 1:**
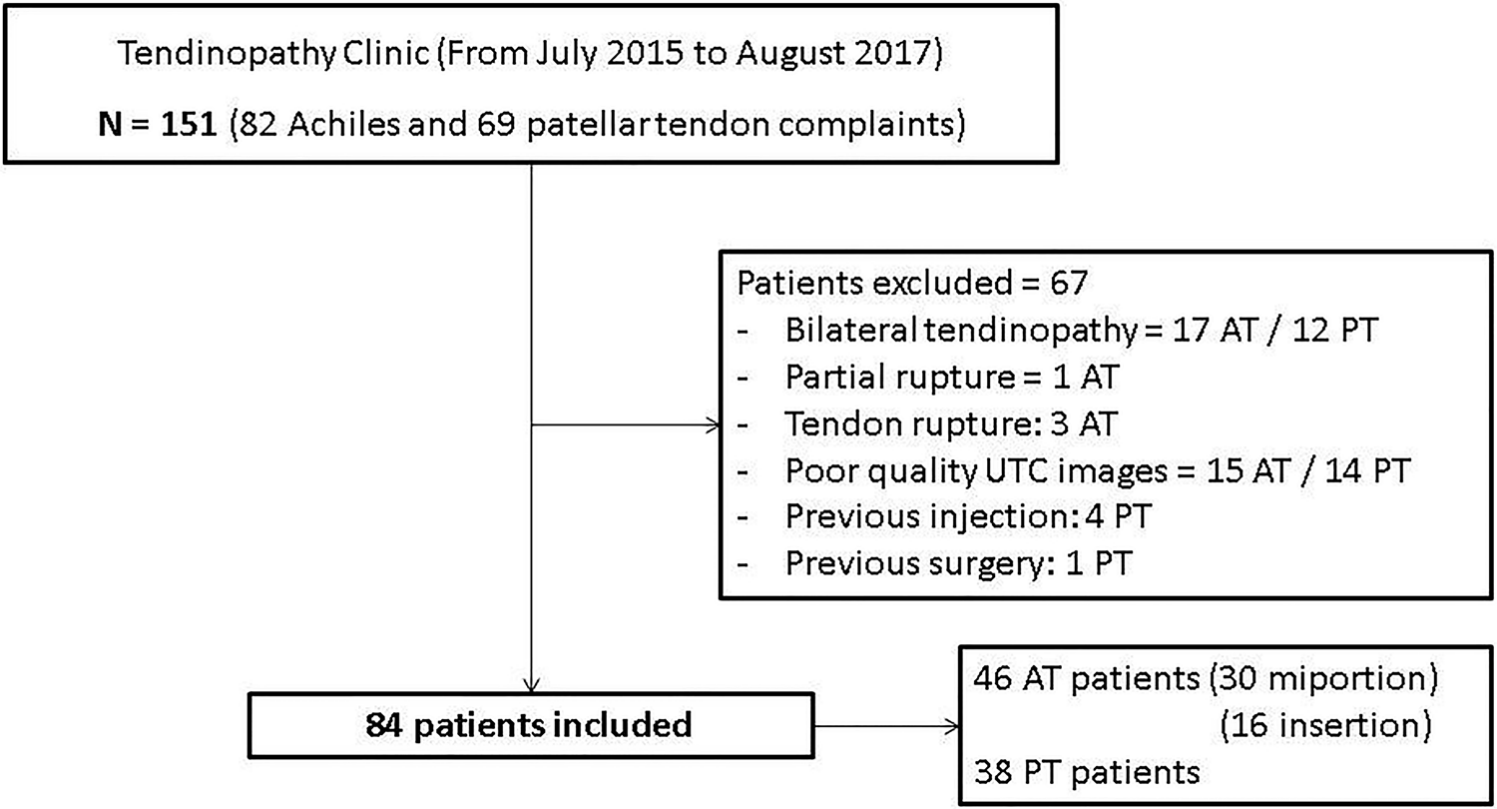
Flow diagram of patients inclusion

After excluding patients based on the exclusion criteria, a total of 84 patients were included and divided into 3 different groups: insertional AT (16 patients), midportion AT (30 patients) and PT (38 patients). The inclusion process is shown in Fig. 1. The control group consisted of 18 Achilles and 25 patellar tendons. The characteristics of the patients and controls are shown in Table 1. Since patients were recruited from a clinic specialized in tendon rehabilitation, 62% of the insertional AT group, 50% of the midportion AT group and 63% of the PT group reported chronic symptoms (longer than 1 year). None of the patients included in this study reported previous complaints in the asymptomatic side.

**Table 1:** Characteristics of the patients and control subjects included in the study

	Tendinopathy patients			Controls	
	Insertional AT (*N* = 16)	Midportion AT (*N* = 30)	PT (*N* = 38)	Achilles tendon (*N* = 18)	Patellar tendon (*N* = 25)
Age (years)	45.0 (15.6)	47.8 (12.0)	30.0 (10.1)	22.9 (3.0)	24.9 (2.6)
Gender					
Male, *N* (%)	13 (81)	17 (57)	34 (89)	8 (44)	8 (32)
Female, *N* (%)	3 (19)	13 (43)	4 (11)	10 (66)	17 (68)

The mean (SD) VISA scores of the insertional AT, midportion AT and PT groups were 46.3 (21.2), 58.8 (28.0), and 49.0 (11.4), respectively.

For the Achilles tendon the MDCs were 3.0, 1.6, 1.7, and 1.1% at insertion and 2.4, 2.1, 1.3, and 0.8% at midportion for the four echo types, respectively. For the patellar tendon the MDCs were 4.0, 3.4, 1.2, and 0.4% for the four echo types, respectively.

**Fig. 2:**
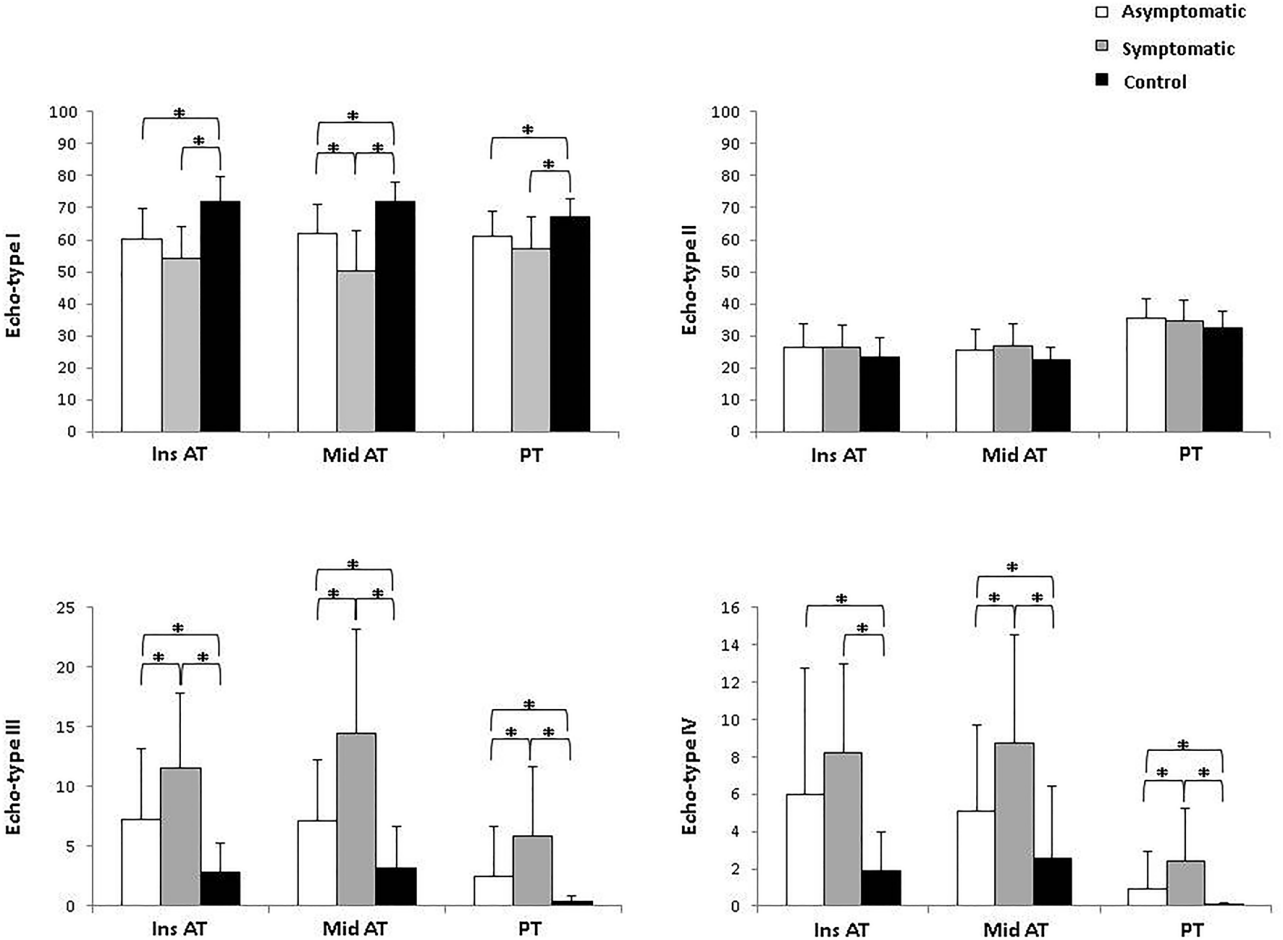
Mean percentages of UTC echo types (I–IV) of patients diagnosed with unilateral AT (insertional or midportion) or PT and controls. *Difference (*p* < 0.01)

For the comparison between asymptomatic and symptomatic sides, different results were found in the different groups. In the insertional AT group a significantly higher percentage of echo type III was observed on the symptomatic side than on the asymptomatic side. In the midportion AT group a significant lower percentage of echo type I and a significantly higher percentage of echo types III and IV were seen on the symptomatic side compared to the asymptomatic side. In the PT group, significantly higher percentages of echo types III and IV were seen on the symptomatic side than on the asymptomatic side. All differences were larger than the minimal detectable changes. Comparing both asymptomatic and symptomatic sides with control subjects, a significantly higher percentage of echo type I and a significantly lower percentage of echo types III and IV were observed in the control group. All differences were larger than the minimal detectable changes. The echo type percentages of each group are shown in Fig. 2.

## Discussion

The most important finding of this study was that also the asymptomatic side of patients diagnosed with unilateral AT or PT showed differences in tendon structure compared to controls. Additionally, the tendon structure of the symptomatic side was significantly different compared to the asymptomatic side. These differences vary between achilles and patellar tendon and, for the achilles tendon, also between the portions of the tendon investigated.

This is the first study to compare the symptomatic side with the asymptomatic side of patients diagnosed with tendinopathy in different tendon portions (insertional or midportion) and different tendon locations (achilles and patella). The symptomatic side, compared to the asymptomatic side, showed different results for each tendon site (insertional or midportion AT) as well as different results for different tendon locations (AT or PT). The results of insertional tendinopathies (insertional AT and PT) might differ from midportion tendinopathy (AT) because the insertional site shows a different tendon structure and mechanical performance than the midportion site [3, 27]. Previous authors showed that the percentage of echo type II is higher at the insertion of the tendon compared to the midportion of the tendon [40]. Further, the different results observed in the PT group—no significant changes in echo type I between symptomatic and asymptomatic sides—when compared to the AT group (where changes were significant) might also be explained by different subject characteristics such as age, gender and physical activity performed [24, 26]. Different tendon properties might also account for the variation in results between AT and PT [10]. A possible explanation for the tendon abnormalities in the asymptomatic side is the cross-transfer effect, commonly observed in muscles [22]. Not only positive effects (e.g., increase in muscle strength) might occur on the contralateral side but also deleterious ones. For example, unilateral electrical stimulation can provoke accumulation of inflammatory cells in the contralateral side [35]. After unilateral electrical stimulation and passive flexion–extension, bilateral changes (increase in tenocyte numbers) in the Achilles tendons of rabbits were observed [2]. A clear explanation is still lacking, but the response is probably mediated by the nervous system [35]. It seems that there is an interhemispheric interaction, demonstrated by the fact that a deconditioning of the affected side exhibits a negative form of cross-education [15].

When comparing symptomatic and asymptomatic tendons with healthy controls it was found that not only symptomatic but also asymptomatic tendon structures are compromised in patients diagnosed with AT (insertional or midportion) or PT. These results are similar to those of Docking et al. [11], who investigated patients with AT (combining insertional and midportion AT). Additionally, studies that investigated tendon structure with conventional US and MRI examination also observed the presence of tendon abnormalities in both the symptomatic side [6, 8] and the asymptomatic side [13, 14]. These findings fit well within the iceberg theory [1] proposed by Abate et al., as well as the continuum model [9] proposed by Cook and Purdam. In both theories, the pathology (tendinopathy) is a continuum. As a consequence of excessive load on the tendon, a sequence of events occurs in the tendon leading first to changes in tendon structure and finally to actual symptoms. These changes occur in both the asymptomatic and the symptomatic side, yet the literature does not offer a clear explanation as to why one tendon turns symptomatic while the other does not [28]. An explanation could be that, despite asymptomatic tendons showing compromised tendon structure, these tendons have insufficient production of nociceptive substances and/or the neural network to reach a threshold to cause pain [29]. UTC might be an important imaging tool to investigate tendon structure and identify the different phases/stages involved in this pathology in both the asymptomatic and the symptomatic side.

This is the first study to compare symptomatic and asymptomatic tendons of three different groups of patients (insertional AT, midportion AT and PT) with young healthy control subjects. Moreover, patients included in the present study were recruited from an outpatient clinic, thus representing a more real, clinical scenario, while previous research focused mainly on male professional athletes. This did limit comparison of the present results with previous findings. The results of this study are not comparable to most previous studies using UTC because a different technique for imaging analysis (window size 17, instead of 25) was used. Using a smaller window size results in a more detailed imaging analysis [40], hence the tendon structure might be better represented in our study.

For clinicians (and patients), identification of imaging abnormalities in the asymptomatic tendon means that the asymptomatic side should not be used as a Ref. [15]. It is nonetheless still relevant to scan the asymptomatic tendon for monitoring purposes, as the presence of imaging abnormalities in the asymptomatic tendon is a risk factor for development of pain and future tendinopathy in that tendon [6, 23, 39]. This is based on studies using conventional US, but there is a dearth of studies using UTC measurement to identify the risk of developing tendinopathy based on an increase of echo types III and IV. A recent study investigating the patellar tendon of young ballet dancers found no significant difference in baseline between the group that did not develop tendon abnormalities after 2 years and the group that developed tendon abnormalities [31]. Other authors found that UTC measurements could not predict Achilles tendinopathy, but the results were based on a population that showed small percentages of echo types III and IV during baseline [41]. More research is needed to confirm if the changes in tendon structure measured with UTC are a risk factor for future tendinopathy. Furthermore, future studies should investigate the effect of therapeutic interventions on the tendon structure of both the symptomatic and asymptomatic sides and assess if these changes influence the development of symptoms and/or outcome of treatment.

Some limitations of this study should be considered when interpreting the results. Other factors that were not investigated in this study could have influenced the outcomes. For example, the effect of aging [21] and/or the use of medication could have had a negative impact on the tendon structure [5, 36]. Since tendinopathy has a multifactorial etiology, we recommend that when examining the tendon structure of patient with tendinopathy, clinicians should take into consideration as much individual patients characteristics as possible.

For clinical practice, the results of this study show that UTC examination should be performed bilaterally in patients with unilateral tendinopathy, to monitor the tendon structure of the symptomatic side and also to identify tendon abnormalities that might be present in the asymptomatic side. Despite the fact that UTC might not be able to predict the development of future symptoms [12], the UTC imaging tool might be used to monitor the changes of achilles and patellar tendons related to the treatment and/or load performed (Rabello et al., personal comunication). Thus, scanning symptomatic and asymptomatic tendons provides additional information to clinicians with respect to future changes in tendon structure. Additionally, clinicians should not use the asymptomatic side as normal reference during their examination.

## Conclusion

Both symptomatic and asymptomatic tendon structures are compromised in patients diagnosed with unilateral insertional or midportion Achilles tendinopathy and patellar tendinopathy compared to young healthy controls. These findings implicate that the asymptomatic side should not be used as normal reference during examination. Still, due to the high risk of developing tendinopathy, clinicians should include examination of the asymptomatic side for monitoring purposes.
